# Seaweed as an Anti-Fat

**Published:** 1884-09

**Authors:** 


### SEA-WEED AS AN "ANTI-FAT."

The use of the variety of sea-weed botanically known as *Fucus vesiculosus* for reducing obesity has obtained recognition in professional circles abroad. Dr. Fairbank writes to the *British Medical Journal* as follows: "More than fifteen years ago, I gave some of the extract in pills (four grains three times a day) to a very corpulent lady, who in three months lost three stone in weight without any change of diet. Since then, I have frequently given it for reducing weight depending on the accumulation of adipose tissue, and have never found it to fail. The solid extract can be easily made into four-grain pills, which must, however, be kept in a stoppered bottle, as they readily absorb moisture from the air. I may say that a patient, who has lately been taking it as an anti-fat, and who always suffered very much from rheumatic pains about the body, has been entirely free from such trouble while she has been taking the extract, a fact which she quite independently noted."

